# The evidence from clinical trials on Gout medicines effect on COVID‐19: A protocol for systematic review and meta‐analysis

**DOI:** 10.1002/nop2.1501

**Published:** 2022-11-28

**Authors:** Ahmad Naoras Bitar, Syed Azhar Syed Sulaiman

**Affiliations:** ^1^ Department of Clinical Pharmacy, Faculty of Pharmacy University of Aleppo Aleppo Syria; ^2^ Department of Clinical Pharmacy, Michel Sayegh College of Pharmacy Aqaba University of Technology Aqaba Jordan; ^3^ Department of Clinical Pharmacy, School of Pharmaceutical Sciences Universiti Sains Malaysia Penang Malaysia

**Keywords:** clinical, clinical decision‐making, clinical effectiveness, clinical trial

## Abstract

**Aim:**

To evaluate the available evidence from clinical trials on the efficacy of gout medicines against COVID‐19.

**Design:**

Systematic review and Meta‐analysis.

**Methods:**

We are systematically searching five databases [PubMed, Embase, CT.gov, ICTRP, CINAHL (EBSCO)]. We are following the PRISMA statement and the EPOC guidelines. The meta‐analysis will be conducted using Revman‐5.4.1 from Cochrane collaboration, UK. This review's protocol was also registered in PROSPERO, University of York, UK (CRD42022299718).

**Results:**

In this meta‐analysis, we plan to give a conclusive overview of the available evidence on the efficacy of the medications used to manage gout in reducing COVID‐19 mortality, ICU admission, ventilation rate and hospitalization duration. If the results were positive, these drugs would greatly add to the scarce treatment options against COVID‐19. Furthermore, these drugs might provide an excellent alternative to inconvenient and expensive drugs. Additionally, most of these drugs have a well‐established safety profile for use during nursing, making them a much safer option for nursing mothers with COVID‐19.

## 1 BACKGROUND

Severe acute respiratory syndrome coronavirus 2 (SARS‐CoV‐2) or better known as coronavirus disease (COVID‐19) is an infectious respiratory disease that could cause acute respiratory distress syndrome (ARDS) in almost one‐third of patients (Tzotzos et al., 2020). The infection of COVID‐19 triggers the release of pro‐inflammatory cytokines and chemokines by stimulating the toll‐like receptors (TLRs) and NOD‐like receptors (NLRs) in immune cells (Khanmohammadi & Rezaei, 2021, p. 19). In severe COVID‐19 patients, the overstimulation NOD‐like receptor family and pyrin‐containing domain 3 (NLRP3) could lead to a cytokine storm that might be life‐threatening (Cron et al., 2021). In addition, the intracellular stress caused by the infection triggers an inflammatory innate immune response by triggering an inflammasome assembly (Gangopadhyay et al., 2022). The inflammasome assembly is initiated by NLRP3 sensing the infectious patterns, NLRP11 interacts with NLRP3 and ASC leading to the inflammasome assembly activation, NLRP3 and ASC polymerization, caspase‐1 activation, pyroptosis and cytokine release (Akkaif, Daud, et al., 2021; Gangopadhyay et al., 2022).

Gout is an inflammatory form of arthritis and it is caused by continuous and persistent hyperuricemia; the elevated serum urate levels lead to the formation and deposition of monosodium urate crystals in and around the joints (Dehlin et al., 2020). Pseudogout is also an inflammatory bone disease, and it is caused by the deposition of calcium pyrophosphate (CPP) crystals in joints (Zamora & Naik, 2022). Both conditions are usually treated with colchicine and immunosuppressant drugs. Colchicine prevents acute gout flares because of its anti‐inflammatory properties (Dalbeth et al., 2014), while allopurinol and febuxostat inhibit xanthine oxidase and thus reduce the synthesis of uric acid (UA) (Strilchuk et al., 2019), febuxostat and allopurinol lower the UA levels and attenuate the expression of an inflammatory marker, monocyte chemoattractant protein (MCP)‐1(Nakagomi et al., 2015). On the other hand, probenecid facilitates the excretion of uric acid by suppressing renal tubular transport (inhibits pannexin 1 channels) and blocking its reuptake (Patel et al., 2021). While, indomethacin inhibits the prostaglandins' synthesis by cyclooxygenase (COX) enzymes, thus reducing the amount of serum inflammatory mediators (Munjal & Allam, 2022).

COVID‐19 infection induces a massive systemic inflammatory response, and the medicines that are used for the treatment of gout display an immunomodulatory behaviour and have anti‐inflammatory properties (Akkaif et al., 2022; Burrage et al., 2020, p. 2). Therefore, the question is, do the drugs used for the treatment of gout reduce the mortality or the severity of COVID‐19 (independently/with concomitant treatment) or not? To answer this question, we have conducted a systematic review and a meta‐analysis of the available data from clinical trials to assess and evaluate the current evidence on the presented topic. Also, this meta‐analysis will give an accurate idea about the used doses for COVID‐19 and whether dose adjustment for severe patients is required or not. Furthermore, COVID‐19 is likely to stay around for long, and identifying the effective drugs in COVID‐19's management is essential to reduce its impact on the general population and the healthcare systems. Therefore, this review aims to evaluate the evidence from clinical trials on the efficacy of gout medicines (methylprednisolone, prednisolone, febuxostat, allopurinol, indomethacin, lesinurad, pegloticase, probenecid and colchicine) against SARS‐CoV‐2.

## 2 METHODS/DESIGN

**FIGURE 1:**
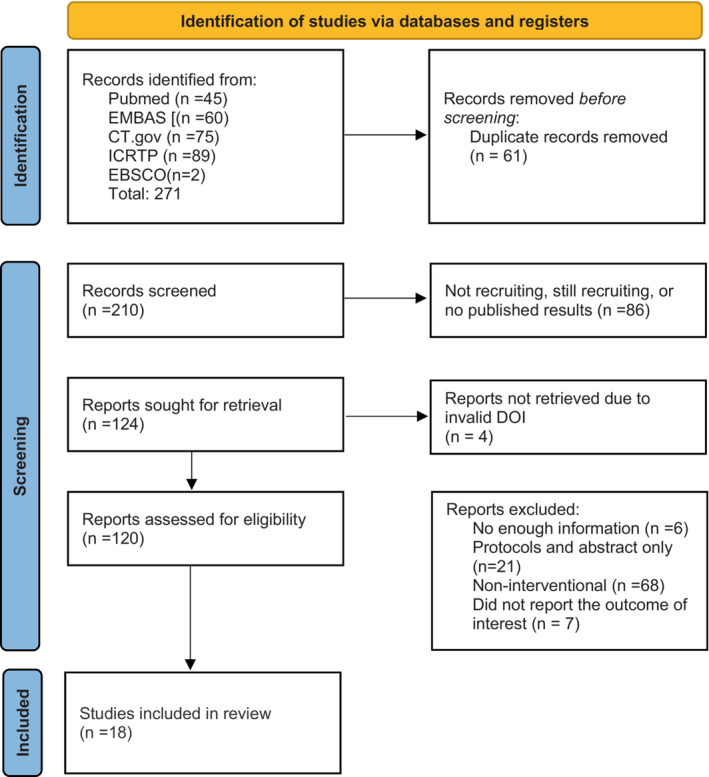
The primary or current PRISMA flow diagram for the systematic review including searches of databases and registers

In this review, we are systematically searching five databases [PubMed, Embase, CT.gov, ICTRP, CINAHL (EBSCO)]. We are following the Preferred Reporting Items for Systematic Reviews and Meta‐Analyses guidelines (PRISMA) statement guidelines on systematic reviews protocols and the Cochrane Effective Practice and Organization of Care (EPOC) guidelines (Figure 1). First: Identification of articles and deleting of duplicates; second: Screening and collections of manuscripts based on the title and abstract. Third: full‐text thorough analysis for the inclusion and exclusion of manuscripts. This review's protocol was also registered in the International Prospective Register of Systematic Reviews, University of York, UK (registration number: CRD42022299718) (Bitar & Sulaiman, 2022a). We will use Cochrane's data extraction sheets for collecting the required information and Cochrane's risk of bias assessment tool to evaluate the included manuscripts (RoB 2 and ROBINS‐I). Furthermore, the meta‐analysis for the included articles will be conducted using Revman‐5.4.1 (using inverse‐variance random effect model) from the Cochrane collaboration. If the meta‐analysis's results show a high heterogeneity, a subgroup analysis will be conducted to identify the sources or the causes of the heterogeneity. In case of some missing or unclear information in the manuscripts, we will request the corresponding authors to provide the required data via email. The manuscripts will be excluded if the authors do not respond in 2 weeks. The sociodemographic characteristics of the included subjects are to be analysed in this systematic review and meta‐analysis (including age, BMI, gender, medical history, severity, comorbidities and received/prescribed medication).

### 2.1 Inclusion criteria

(1) Clinical trials (randomized controlled tails and non‐randomized controlled trials) that have included COVID‐19 patients receiving gout medications (oral or IV) and compared them to patients receiving standard of care or placebo. (2) Analysed gout treatment among COVID‐19 patients. (3) Have presented the results in OR (odds ratio) or RR (risk ratio). (4) Have reported on the following outcomes among indoor and outdoor patients, or both: mortality and hospitalization. (5) Published in the English language. (6) Published in the last 2 years.

### 2.2 Exclusion criteria

(1) Review articles, editorial articles, abstracts, congress abstracts, case reports, case series and current clinical trials. (2) Grey literature materials and republished manuscripts. (3) Incomplete or current research. (4) End of life or palliative care studies. (5) Manuscripts that are not available in full text.

### 2.3 Participants of the studies participants

Adult patients with a confirmed diagnosis of SARS‐CoV‐2.

### 2.4 Type of intervention

Gout treatment with or without other COVID‐19 treatment.

### 2.5 Comparator(s)/control

Standard of care or COVID‐19 treatment without any gout medicine.

### 2.6 Main outcome

The primary outcome is to find out whether gout treatment or medicines have reduced the mortality rates among COVID‐19 patients or not.

### 2.7 Measures of effect

The effect size of interest is the pooled odds ratios or risk ratios of the included studies.

### 2.8 Additional outcome

Secondary outcomes include ICU admission, duration of hospitalization, acute respiratory distress syndrome, required ventilation and clinical evaluation in nursing mothers.

### 2.9 Search strategy

(((((“colchicine”[All Fields]) AND (“covid 19”[All Fields])) OR (“mortality”[All Fields])) OR (“length of stay”[All Fields])) OR (“icu admission”[All Fields])) OR (“complication”[All Fields]). (((((“Allopurinol”[All Fields]) AND (“covid 19”[All Fields])) OR (“mortality”[All Fields])) OR (“length of stay”[All Fields])) OR (“icu admission”[All Fields])) OR (“complication”[All Fields]). (((((“Febuxostat”[All Fields]) AND (“covid 19”[All Fields])) OR (“mortality”[All Fields])) OR (“length of stay”[All Fields])) OR (“icu admission”[All Fields])) OR (“complication”[All Fields]). (((((“Indomethacin”[All Fields]) AND (“covid 19”[All Fields])) OR (“mortality”[All Fields])) OR (“length of stay”[All Fields])) OR (“icu admission”[All Fields])) OR (“complication”[All Fields]). (((((“Lesinurad”[All Fields]) AND (“covid 19”[All Fields])) OR (“mortality”[All Fields])) OR (“length of stay”[All Fields])) OR (“icu admission”[All Fields])) OR (“complication”[All Fields]). (((((“Pegloticase”[All Fields]) AND (“covid 19”[All Fields])) OR (“mortality”[All Fields])) OR (“length of stay”[All Fields])) OR (“icu admission”[All Fields])) OR (“complication”[All Fields]). (((((“Probenecid”[All Fields]) AND (“covid 19”[All Fields])) OR (“mortality”[All Fields])) OR (“length of stay”[All Fields])) OR (“icu admission”[All Fields])) OR (“complication”[All Fields]). (((((“Prednisolone”[All Fields]) AND (“covid 19”[All Fields])) OR (“mortality”[All Fields])) OR (“lengtht of stay”[All Fields])) OR (“icu admission”[All Fields])) OR (“complication”[All Fields]). (((((“Methylprednisolone”[All Fields]) AND (“covid 19”[All Fields])) OR (“mortality”[All Fields])) OR (“lengtht of stay”[All Fields])) OR (“icu admission”[All Fields])) OR (“complication”[All Fields]). Furthermore, the hand picking method was used to pick articles that might be eligible for this review from the references list of the included manuscripts. Two reviewers ran the search results separately and independently evaluated the included articles. The screening for articles was made in three phases according to PRISMA criteria.

### 2.10 Ethics

No formal research ethics committee approval is required since the review's synthesis will be based on published data.

## 3 DISCUSSION

In this review and meta‐analysis, we will investigate various medicines that are being used for the treatment and management of gout and their impact on SARS‐CoV‐2 patients. The main outcome in this research work will be the mortality rate; however, we are planning to examine the duration of hospitalization, the need for ICU and the need for mechanical ventilation as secondary outcomes. The medicines used to manage gout flares are commonly available, effective and affordable; these drugs might be of great use in the fight against COVID‐19 because of their anti‐inflammatory effect and well‐established safety profiles.

A few clinical trials have investigated the effect of the medicines used in gout treatment against COVID‐19. Some studies have investigated the efficacy of colchicine (Absalón‐Aguilar et al., 2022; Akkaif, Ng, et al., 2021; Deftereos et al., 2020), and others have tested the impact of XOIs like allopurinol and COX enzyme inhibitors on COVID‐19 patients (Al‐Kuraishy et al., 2022), and one study has found that Probenecid has potential clinical benefits in the management of COVID‐19 (Murray et al., 2021). Corticosteroid drugs (methylprednisolone and prednisolone) effect on the severity of the disease was also investigated in a few clinical trials because of their immunosuppressant effect (Barros et al., 2021; Bitar & Sulaiman, 2022b; Ghanei et al., 2021).

Most of the included drugs have a well‐established safety profile for use during nursing, making them a much safer option for nursing women with COVID‐19. Although colchicine was present in the milk sera of nursing mothers, its concentration was negligible, and it was safe to use during nursing (Ben‐Chetrit, 2018), and no side effects or long‐term effects were observed among the breastfeeding infants (Herscovici et al., 2015). According to the drug and lactation database in the US's national library of medicine, several reports and studies for breastfeeding mothers using prednisone or methylprednisolone showed no side effects on the infants. However, medium to large doses of depot corticosteroids injected into joints had been reported to cause a temporary reduction of lactation (“Methylprednisolone,” 2006; “Prednisone,” 2006). Febuxostat is not contraindicated during breastfeeding because it has more than 99% plasma protein binding rate with an oral bioavailability of 50% only, which makes the concentration that might appear in the breast milk negligible (Anderson, 2021). According to the Drugs and Lactation Database in the United States, the levels of indomethacin were undetectable in the vast majority of mothers' breast milk after doses between 75 mg–300 mg daily; however, the excretion of the glucuronide metabolite into milk was not measured, and it could be absorbed as indomethacin by a new born (“Indomethacin,” 2006). According to Philip O. Anderson, allopurinol was undetectable in the infant's plasma after the mother was prescribed an oral 300 mg/daily dose for 4 weeks. Still, the infant had around one‐third of maternal oxypurinol levels but showed no observable side effects or changes in the clinical chemistry or the haematology values (Anderson, 2021). Despite the fact that the detected concentration of probenecid in the mother's milk was acceptably low (145 mcg/kg) after she was given 2 g of probenecid, the infant had some undesirable side effect like green liquid stool, severe diarrhoea, discomfort and crying (Anderson, 2021). However, the researchers attributed these side effect to cephalexin because the mother was taking cephalexin too. Unfortunately, the safety profile for lesinurad and pegloticase during nursing was not established yet.

This systematic review and meta‐analysis will provide a conclusive overview of the available evidence. If the results were positive, these drugs would greatly add to the scarce treatment options available against COVID‐19. Furthermore, these drugs might provide a great alternative for inconvenient and expensive drugs. Additionally, most of these drugs have a well‐established safety profile for use during nursing, making them a much safer option for nursing mothers with COVID‐19. We project the review and meta‐analysis to be ready by November to December of 2022, depending on the response from researchers about missing information in their published reports. The findings and results from this research project are to be published in a peer‐reviewed journal.

## FUNDING INFORMATION

This study received no funding or sponsorship from any entity.

## CONFLICT OF INTEREST

None.
